# Blood Count and Renal Functionality Assessments in the Emergency Section Disclose Morbidity and Mortality in Omicron COVID-19 Patients: A Retrospective Study

**DOI:** 10.3390/clinpract14030055

**Published:** 2024-04-23

**Authors:** Eqrem Rusi, Fiorenza Pennacchia, Wael Abu Ruqa, Maria Antonella Zingaropoli, Patrizia Pasculli, Giuseppina Talarico, Giuseppe Bruno, Christian Barbato, Antonio Minni, Luigi Tarani, Gioacchino Galardo, Francesco Pugliese, Marco Lucarelli, Maria Rosa Ciardi, Luigi Meucci, Giampiero Ferraguti, Marco Fiore

**Affiliations:** 1 Department of Human Neuroscience, Sapienza University of Rome, 00185 Rome, Italy; 2 Department of Sensory Organs, Sapienza University of Rome, 00185 Rome, Italy; 3 Department of Public Health and Infectious Diseases, Sapienza University of Rome, 00185 Rome, Italy; 4 Institute of Biochemistry and Cell Biology (IBBC-CNR), c/o Department of Sensory Organs, Sapienza University of Rome, 00185 Rome, Italy; 5 Division of Otolaryngology-Head and Neck Surgery, ASL Rieti-Sapienza University, Ospedale San Camillo de Lellis, 02100 Rieti, Italy; 6 Department of Maternal Infantile and Urological Sciences, Sapienza University of Rome, 00185 Rome, Italy; 7 Medical Emergency Unit, Sapienza University of Rome, 00185 Rome, Italy; 8 Department of Anesthesiology Critical Care Medicine and Pain Therapy, Sapienza University of Rome, 00185 Rome, Italy; 9 Department of Experimental Medicine, Sapienza University of Rome, 00185 Rome, Italy; 10 Directorate Social and Welfare Statistics, ISTAT, 00184 Rome, Italy

**Keywords:** SARS-CoV-2, mortality, morbidity, biomarkers, variant, blood analyses, Omicron

## Abstract

**Background**: SARS-CoV-2 is the coronavirus responsible for the COVID-19 pandemic. Even though we are no longer in a pandemic situation, people are still getting infected, some of them need hospitalization and a few of them die. **Methods**: We conducted a retrospective study including 445 patients who accessed the Emergency Section of Policlinico Umberto I, Rome, Italy, where they had routine blood exams. In this study, we focused on the complete blood count, serum creatinine and azotemia. The data were analyzed using ANOVA, Spearman correlation and ROC analyses. They were divided into four groups based on their clinical outcomes: (1) the *emergency group* (patients who had mild forms and were quickly discharged); (2) the *hospital ward* group (patients who were admitted to the emergency section and were then hospitalized in a COVID-19 ward); (3) the intensive care unit (*ICU*) group (patients who required intensive assistance after the admission in the emergency section); (4) the *deceased* group (patients who had a fatal outcome after admission to the emergency section). **Results**: We found significant changes for creatinine, azotemia, hematocrit, mean corpuscular hemoglobin concentration, basophils, monocytes, red blood cell distribution width, hemoglobin, hematocrit and red blood cell numbers using ANOVA according to their clinical outcomes, particularly for the deceased group. Also, we found linear correlations of clinical outcomes with eosinophils, hemoglobin, hematocrit, mean corpuscular hemoglobin concentration, lymphocyte, neutrophil, platelet and red blood cell number and red blood cell distribution width. **Conclusions**: This study discloses an early association between “classical” routine blood biomarkers and the severity of clinical outcomes in Omicron patients.

## 1. Introduction

Since early 2020, the world has been fighting COVID-19 (coronavirus disease 2019), the respiratory disease caused by SARS-CoV-2 (severe acute respiratory syndrome coronavirus-2) [1,2,3,4]. The virus belongs to the coronaviridae subfamily, more specifically in the *Betacoronavirus* genera, and has a positive-sense single-stranded RNA genome [5,6,7].

The first cases of COVID-19 date back to late 2019 in the city of Wuhan (Hubei Province, China), but the origin of the virus is still unknown, although it is thought of as a natural evolution from an animal host to a human one. In favor of this theory, the fact is that the first few cases were linked to the Seafood Wholesale Market, where live animals were sold [7,8]. From the report of the first few cases until 18 October 2023, there were 696,695,527 registered cases worldwide, with 6,927,179 deaths [9], while in Italy, there were 26,168,412 registered cases, with 192,013 deaths [10]. The transmission of the virus primarily occurs through respiratory droplets produced when an infected person coughs, sneezes, talks or breathes. These droplets can be inhaled by people nearby or can contaminate surfaces, where viruses can survive for several hours or even days [11,12,13,14]. Subsequently, SARS-CoV-2 enters the host cell through different mechanisms, inducing different clinical pictures, from asymptomatic to Acute Respiratory Distress Syndrome and Multi-Organ Injury [14]. 

Like any other virus, the coronavirus tends to mutate [15,16,17]. During the spreading of the infection, several variants of the virus emerged, further complicating the management of the pandemic. Each SARS-CoV-2 variant differs from the original strain due to these mutations, which can affect the virus transmissibility, the disease severity and the immune response [18]. Recently, a group of authors published a study, which demonstrates that isolation measures during the pandemic drove faster and more transmissible SARS-CoV-2 variants [19]. With the outbreak of new variants, WHO experts created a classification that divided the variants into different groups. The two most important are the VOCs (variants of concern) and VOIs (variants of interest) [20]. At the moment, there are no variants that meet VOC criteria [21].

Many previous VOCs are the Delta variant (B.1.617.2), which originated in India, and the Omicron variant originated in South Africa and was first identified in November 2021. The Delta variant has been associated with high transmissibility and rapidly spread in many countries. This increased transmissibility led to a significant rise in cases and posed an additional challenge to healthcare systems worldwide [15,16,17]. The Omicron variant, on the other side, was associated with less severe disease but had an increased transmissibility. One of the main concerns regarding the Omicron variant is its high genetic mutability. It carries a significant number of mutations in its genetic material, particularly in the spike protein gene that the virus uses to enter human cells. Among these, some are similar to those found in other variants of concern, such as Beta, Gamma and Delta. However, the combination and widespread presence of these mutations are what make the Omicron variant unique and raise doubts about its potential ability to evade immunity [22]. 

Currently, there are few data available on Omicron’s predictability regarding mortality and morbidity. As has been carried out for other COVID-19 variants [23,24,25,26], we previously analyzed specific COVID-19 biomarkers from routine blood tests conducted on COVID-19 Omicron patients at the emergency section level [23,25]. In this study [25], we demonstrate that troponin-T (TnT), fibrinogen (FBG), glycemia, C-reactive protein (CRP), lactate dehydrogenase (LDH), albumin, D-dimer, myoglobin (MGB) and ferritin, for both men and women, may predict, at the level of the emergency section, lethal outcomes. Compared to previous Delta COVID-19 parallel emergency patterns of prediction in the *emergency* room, we discuss that Omicron-induced changes in TnT and albumin may be considered early predictors of severe outcomes. In our cohort of patients, we show that the main percentage of unvaccinated women was in the *deceased* group [25]. We also show LDH potentiation in unvaccinated patients. Surprisingly, vaccinated patients had higher TnT values when compared to unvaccinated individuals. As for the COVID-19 vaccine’s effectiveness against Omicron, in our cohort of patients, we disclose that primary immunization with more than two doses significantly increased protection.

Thus, the *main aim* and *novelty* of this study were to investigate the “classical” routine blood biomarkers in the same cohort of patients to correlate these data with the severity of their clinical outcomes. We gathered data from 445 COVID-19 clinical records from the Emergency Room of “Policlinico Umberto I”, at the University Hospital of Sapienza University of Rome. According to their clinical outcomes, the 445 patients were divided into four groups: (1) the *emergency* group (patients who had mild forms and were quickly discharged); (2) the *hospital ward* group (patients who were admitted to the emergency section and were hospitalized in a COVID-19 ward); (3) the intensive care unit (*ICU*) group (patients who required intensive assistance after admission to the emergency section); (4) the *deceased* group (patients who had a fatal outcome after admission to the emergency section). 

In this study, in particular, we analyzed the possible correlations of creatinine, azotemia, blood urea nitrogen, red blood cells (RBCs), hemoglobin (Hb), hematocrit (Hct), mean corpuscular volume (MCV), mean corpuscular hemoglobin (MCH), mean corpuscular hemoglobin concentration (MCHC), red blood cell distribution width (RDW), monocytes, eosinophils, basophils, white blood cells (WBCs), neutrophils, lymphocytes, platelets (PLTs) and plateletcrit (PCT) with the clinical outcomes of the patients.

## 2. Materials and Methods

### 2.1. Participants’ Selection and Study Design

This retrospective study is based on the clinical records of 445 COVID-19 patients who accessed the emergency unit of the Sapienza University Hospital “Policlinico Umberto I” of Rome, Italy, from 1 February 2022 to 31 March 2022. Of the 445 patients, 130 (29.2%) were not vaccinated. The clinical records of the patients were also utilized for other parameters in a previous study [25].

**Figure 1:**
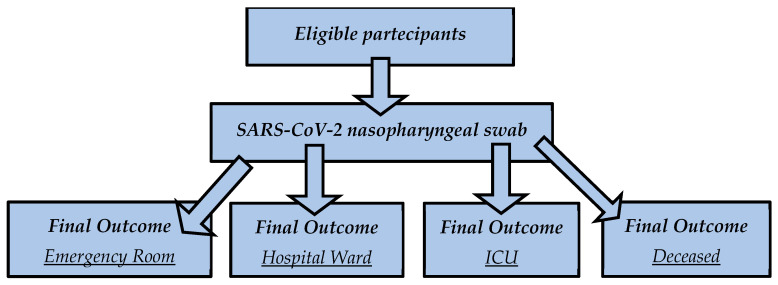
Participants flow diagram according to their outcome.

According to our previous study sharing the same cohort of patients [25], we divided the patients into four groups according to their outcome (Figure 1). Starting from the first one and moving to the last, the outcome worsens:

1. The first group (180, M = 76; F = 104), also called the “*emergency* group”, included those patients who entered the emergency room and were discharged shortly after because they did not show severe symptoms
2. The second group (205, M = 105; F = 100), also called the “*hospital ward* group, *ward* in the text and figures”, included those patients admitted to the emergency room and then transferred to a COVID-19 ward and dismissed afterward.
3. The third group (25, M = 14; F = 11), or the “*ICU* group”, included those who, after admission to the hospital ward, were transferred to the COVID-19 intensive care units and survived (*ICU* group).
4. In the fourth group (35, M = 23; F = 12), some patients had a fatal outcome (in the emergency room, in the hospital ward or the ICU). We called this group the “*deceased* group”.

The diagnosis of SARS-CoV-2 infection was based on a positive result from real-time reverse-transcription polymerase chain reaction (RT-PCR) testing of nasopharyngeal-swab specimens. Patients who tested positive in the molecular test during recovery were transferred to the hospital’s COVID-19 wards. 

The University Hospital ethical committee approved this retrospective study (Ref. 6536), and all the study procedures followed the Helsinki Declaration of 1975, as revised in 1983, for human rights and experimentation.

### 2.2. Patient’s Medical Records

For each eligible patient, we extracted information from their medical records, such as demographic characteristics (age and sex), vaccination, symptoms, comorbidities and laboratory analytical results. The results of the available laboratory tests were collected when patients were initially admitted to the *emergency* unit. Table 1 shows the considered analyses and the number of patients analyzed for each test concerning the total subjects in the four groups.

### 2.3. Laboratory Examination

The patients’ peripheral blood was collected in BD vacutainer^®^ tubes for blood testing at the entrance of the hospital ward. The additives present in vacutainers were EDTA or sodium citrate as anticoagulants and separating gel for serum samples. Coagulation parameters were analyzed with a BCS XP System automatic hemostasis analyzer (Siemens Healthcare, Erlangen, Germany). PLT (reference range: 150–450 × 10^3^/μL), RBC (reference range number 3.5–5.1 × 10^6^/μL for women, 4.3–5.9 × 10^6^/μL for men) and WBC (reference range: 4.4–11.3 × 10^3^/μL) were considered in this study. PCT and Hb (reference range: 12.2–15.3 g/dL for women and 13.5–16.5 g/dL for men) were determined using ADVIA 2120i Hematology System (Siemens Healthcare, Erlangen, Germany). Serum biomarkers (azotemia and creatinine) were measured by standard colorimetric and enzymatic methods on a Cobas C 501 analyzer, with reagents supplied by Roche Diagnostics GmbH (Mannheim, Germany).

### 2.4. Statistical Analysis

According to methods previously described [27,28], data were analyzed to assess normality via Pearson’s chi-squared test. Two-way analysis of variance (ANOVA) (*emergency* vs. *ward* vs. *ICU* vs. *deceased* and men vs. women) was used to analyze the laboratory parameters and the vaccination data. Post hoc comparisons were carried out by using Tukey’s HSD test. The Spearman correlation test was used to investigate the correlation between the laboratory data and the age of the patients [29]. A receiver operating characteristic (ROC) analysis was performed to measure the diagnostic/predictive accuracy of each variable [27]. All analyses were performed using Epitools by Ausvet https://epitools.ausvet.com.au/roccurves, last access, 20 April 2024 (Australia) and StatView 5.0 (Abacus Corporation, Baltimore, USA).

## 3. Results

We gathered all patients’ COVID-19 manifestations and their clinical conditions from the clinical records of the emergency room. All data, divided into each group and sex, are shown in Table 2.

**Figure 2:**
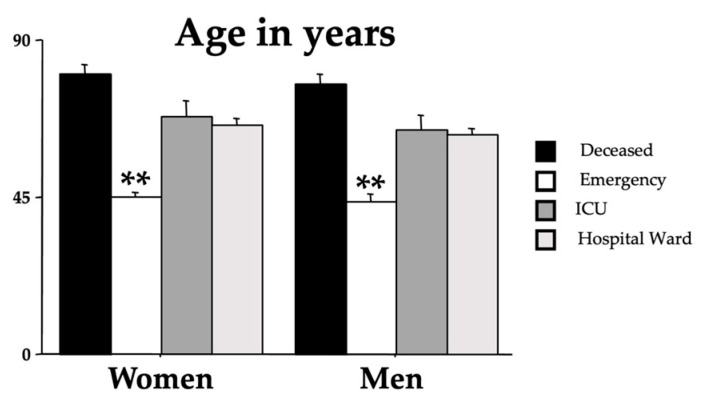
Mean age in years of the recruited individuals for each group divided by sex. The error bars indicate pooled standard error means (SEMs) derived from the appropriate error mean square in the ANOVA. The asterisks (** *p* < 0.01) indicate the post hoc differences between the emergency group and all the other groups.

ANOVA analyses were performed to assess differences in the age and sex of the different outcome groups. Figure 2 shows the influence of age on the outcomes (F(3,437) = 62.82, *p* < 0.001). Indeed, younger patients had a more favorable outcome, while there was no sex effect on the outcome (F(1,437) = 1.18, *p* = 0.277). No interaction outcome x sex was found (F(3,437) = 0.11, *p* = 0.951).

**Figure 3:**
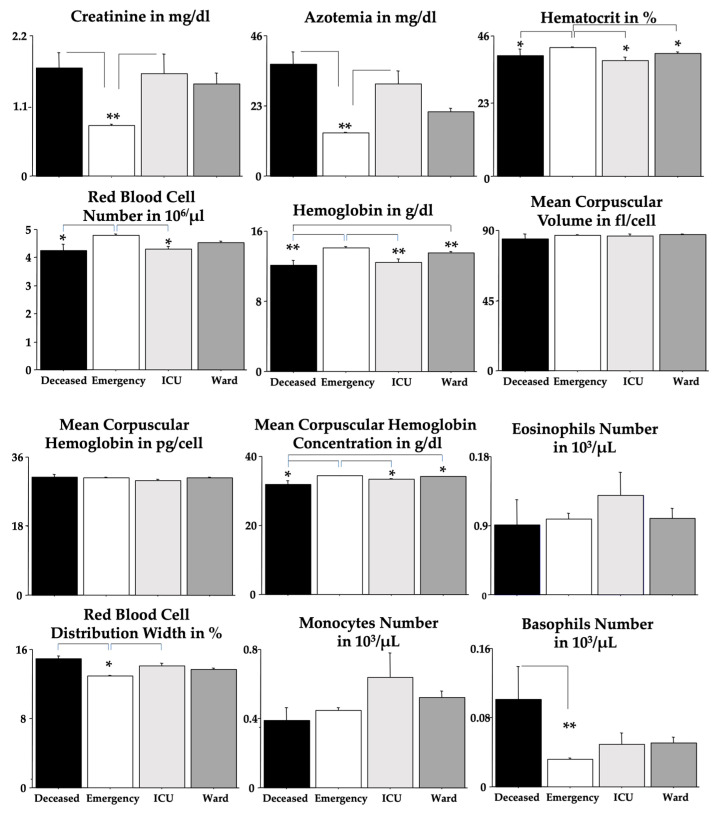

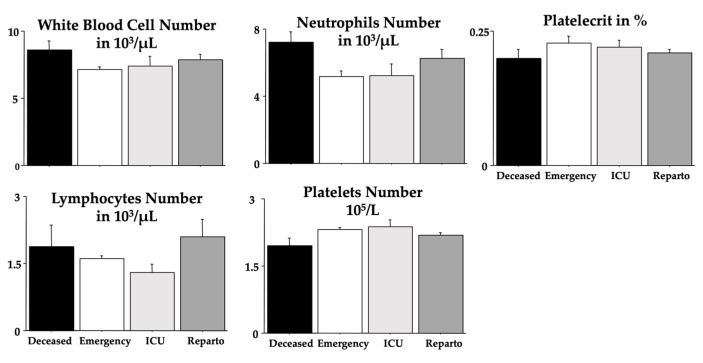
Blood parameters were analyzed by using ANOVA. The error bars indicate pooled standard error means (SEMs) derived from the appropriate error mean square in the ANOVA. The asterisks (** *p* < 0.01; * *p* < 0.05) indicate post hoc differences between groups.

Each blood parameter was analyzed by using an ANOVA test for each group (Table 3). Figure 3 shows these findings but without the sex effect. We found significant elevations due to severe outcomes in creatinine, azotemia, RDW and basophils but significant diminutions in RBC, Hb, Hct and MCHC when compared to the *emergency* group. Post hoc comparisons are shown in the figures as asterisks and lines. 

As expected, for RBC, Hb and Hct, we found significant differences between men and women (Table 3); unexpectedly, we found a sex-linked difference in PLT. ANOVA presented statistical interactions between “outcomes” and “sexes” for MCV and MCHC. Quite interestingly, no differences between outcomes were revealed for MCH, MCV, eosinophils, lymphocytes, neutrophils, PCT, PLT and WBC. 

Table 4 and Table 5 show the ROC data for creatinine, azotemia, RBC, Hb, Hct, MCV, MCH, MCHC, RDW, monocytes, eosinophils, basophils, WBC, neutrophils, lymphocytes, PLT and PCT. The area under the curve (AUC) scores for creatinine, azotemia and RDW unveiled the highest values (in bold in Table 4) in the deceased group.

The positive predictive values (PPVs) in the deceased, ICU and hospital ward groups and the negative predictive values (NPVs) in the emergency group based on the reference range values for creatinine, azotemia, RBC, Hb, Hct, MCV, MCH, MCHC, RDW, monocytes, eosinophils, basophils, WBC, neutrophils, lymphocytes, PLT and PCT are shown in Table 5. In the deceased group, the highest PPV scores were shown for the eosinophils (in bold in the table). No significant PPV scores were found for both the ICU and ward groups. Quite surprisingly, significant NPV scores (in bold in the table) in the emergency group were found for all the analyzed blood parameters but not for creatinine, RBC (both men and women), PCT, lymphocytes and, as expected, eosinophils. 

Table 6 shows the Spearman correlations for the blood biomarkers and the patients’ outcomes. As expected, significant correlations (in bold in the table) were revealed for creatinine, azotemia, RBC, Hb, Hct, MCHC, RDW, eosinophils, lymphocytes and PLT. However, no significant correlations were found for MCV, MCH, monocytes, basophils, WBC, neutrophils and PCT. 

To disclose whether or not the age effect could have impacted the blood parameters of the patients with the worst outcome, we provide further Spearman correlations but only for the *deceased* group (shown in Table 7). Indeed, quite interestingly, positive correlations were found for WBC and neutrophils but not for lymphocytes. No correlations in *deceased* men or women were found for RBC, HB, HCT and PLT, blood parameters with significant sex effects in the ANOVA (see Table 3).

**Figure 4:**
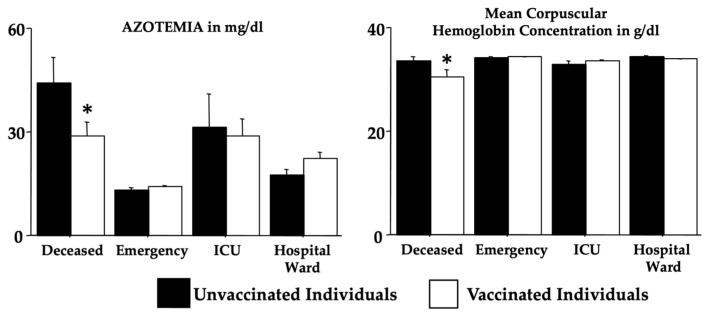
Vaccination effects on azotemia and mean corpuscular hemoglobin concentration (see Table 8). The error bars indicate pooled standard error means (SEMs) derived from the appropriate error mean square in the ANOVA. The asterisk (* *p* < 0.05) indicates post hoc differences between vaccinated and unvaccinated individuals in the *deceased* group.

Table 8 shows the vaccination effects via two-way ANOVA (in the absence of a sex effect) on the selected analyzed blood biomarkers. Data revealed an interaction of Omicron morbidity x vaccination for creatinine, azotemia, Hb, MCV, MCH and MCHC due to differences between groups and an effect of vaccination for MCHC (*deceased*, *emergency*, *ICU* and *ward* × vaccinated and unvaccinated individuals—please see F, dF and *p* on Table 8). Notably, Figure 4 shows the post hoc comparisons according to the mortality for azotemia and MCHC. Indeed, for azotemia, vaccination for individuals in the *deceased* group appears to counteract the marked elevation, whereas for MCHC, vaccination appears to aggravate the condition (both compared to individuals in the *emergency* group).

## 4. Discussion

In this retrospective research on Omicron COVID-19 patients, we show, for the first time, to the best of our knowledge, by analyzing the routine blood analyses normally carried out on the patients attending the emergency room of the Sapienza University Hospital of Rome, that some routine blood parameters could have provided early reliable information on the Omicron COVID-19 outcome.

We disclosed early common blood data in a cohort of 445 patients who experienced different Omicron outcomes, i.e., facing a fatal outcome, attending the ICU but surviving or attending a hospital ward or only the emergency room. According to this group differentiation (*emergency* vs. *ward* vs. *ICU* vs. *deceased*), we evaluated the clinical records of Omicron patients who entered the emergency unit. 

Patients in the *emergency* group were then discharged since they did not display severe symptoms and signs. Patients in the *ward* group attended the dedicated COVID-19 hospital room to be quickly released without significant concerns. Regrettably, other Omicron COVID-19 patients (in the *ICU* and *deceased* groups) experienced more severe infection effects, with or without a lethal outcome.

We found that Omicron COVID-19 patients who later developed a deadly outcome had early gross changes in routine blood analyses. Indeed, ANOVA investigations showed that creatinine, azotemia, RDW and basophils were strongly potentiated in *deceased* Omicron COVID-19 patients if compared to the *emergency* group. In contrast, RBC, Hb, Hct and MCHC values were markedly decreased in *deceased* Omicron COVID-19 patients if compared to patients in the *emergency* group. ROC data obtained by emergency room blood routine analyses crucially extended these ANOVA findings, indicating that changes in creatinine, azotemia and RDW can be considered early indicators of severe Omicron COVID-19 morbidity and mortality [25]. As for the possible predictive value of laboratory markers, PPV data also showed that striking changes in the presence of blood basophils could indicate plain Omicron COVID-19 morbidity and mortality, whereas blood values inside normality ranges for azotemia, Hb (for both men and women), Hct, MCV, MCHC, RDW monocytes, basophils, WBC, neutrophils and PLT represent non-severe Omicron COVID-19 morbidity [25]. 

We also found that vaccination could have influenced the levels of azotemia and MCHC in individuals in the deceased group but with quite different trends. The effect of vaccination, in this cohort of patients, was analyzed in our previous study, dealing with different biochemical parameters [25]. In particular, we found that the highest percentage of unvaccinated women was in the deceased group. Unvaccinated individuals also showed a significant elevation in LDH, particularly in the deceased, ICU and hospital ward groups [25]. Intriguingly, vaccination, when calculated as the number of doses, revealed that the highest number of vaccine doses was disclosed in the emergency group, representing better protection against Omicron-associated morbidity and mortality [25]. 

The COVID-19 pandemic, caused by the novel coronavirus SARS-CoV-2, brought about significant changes in various aspects of healthcare. Among these, routine blood analyses have faced notable implementations and new patterns of interpretation. Routine blood analyses played a crucial role in revealing alterations that prompted clinicians to consider the possibility of COVID-19 infection. The original insurgence of COVID-19 was associated with various hematological abnormalities [30,31,32]. Patients with severe infections often exhibit lymphopenia, thrombocytopenia and increased levels of inflammatory markers. Moreover, the virus is known to induce a hypercoagulable state, leading to an increased risk of thromboembolic events [33,34,35]. Abnormal clotting parameters may be observed in routine blood tests, necessitating careful monitoring and intervention to prevent complications.

The emergence of the Omicron variant of the SARS-CoV-2 virus has raised other, but minor, concerns globally due to its high transmissibility and potential impact on public health [36,37]. As for the Omicron-induced hematological changes, one consistent finding in individuals infected with the Omicron variant is notable alterations in lymphocyte and PLT counts [38,39]. Lymphocytes play a crucial role in the body’s immune response, and their reduction may indicate the severity of the infection or the impact of the variant on immune cell populations [38,39].

Further, data also suggest that platelet counts may be affected by the Omicron variant [38,39]. Thrombocytopenia (reduced platelet levels) or thrombocytosis (elevated platelet levels) could occur, necessitating careful monitoring and management to address potential complications related to blood clotting [40,41,42].

Our patients showed mild neutrophilia and a generally conserved lymphocyte count. The interesting fact is that the lymphopenia was accentuated in the *ICU* group, while the *deceased* group tended to have a normal count but greater than that in the *emergency* group. Perhaps these differences are a result of the evolution of the virus [43,44]. 

Through this study, we confirm and extend what was previously known for Delta COVID-19 [45]. The platelet count can, indeed, discriminate between patients who will undergo a more severe illness, especially those who will not survive the disease compared with patients with a mild course.

Previous studies showed that the eosinophil count is reduced in COVID-19 patients and, afterwards, is restored to normal if the patient has improved while continuing to decrease in those without an improvement [46]. In contrast, our study did not register a marked eosinophil reduction, but *ICU* patients had a peak in the eosinophil count. We also suggest that this modification is due to COVID-19 variants and a possible peculiar Omicron characteristic [43,44].

Also, the count of basophils is normally decreased in COVID-19 patients [47]. Our patients showed a similar trend. An interesting finding was a difference between the *deceased* group and the other groups. Despite this, within the normality range, we found a marked difference between the patients with the worst outcomes compared to those with the best outcomes.

Even though the COVID-19 emergency has finished, SARS-CoV-2 continues to infect and replicate. In doing so, it still poses a threat to health systems around the world. Nowadays, both people and scientific community alerts are lower, mainly because the mortality level has drastically reduced; nonetheless, every day, people still die from COVID-19 [15]. Alterations in the complete blood count are known to be present in patients with COVID-19 [48,49], but only relatively few studies investigated the possibility of identifying these alterations as prognostic factors.

The strength of this study lies in the classification of Omicron COVID-19 individuals according to their outcomes. The present retrospective investigation focused on the levels of *(i)* blood biochemical parameters, especially cellular parameters, with *(ii)* the aim to predict severe COVID-19 outcomes early by comparing four different groups of Omicron patients. To disclose severe outcomes, analogous investigations were carried out but with groups of patients and other experimental schedules. Typically, the main criteria previously used were oxygen saturation levels, fever, age, respiratory rate, respiratory distress, the presence of bilateral and peripheral ground-glass opacities and arterial blood oxygen partial pressure [50,51,52].

This work has some limitations. The nature of this study leads to hypotheses that, indeed, cannot be expanded to other population cohorts unless validated prospectively. Many factors can influence the outcomes of COVID-19 patients, starting from genetic predisposition and individual lifestyles to the pre-existent disease conditions of the recruited patients. An important limiting factor is the scarce information about the patients’ vaccination status. In our previous work, we showed how difficult it is to obtain clear information about the number of vaccinations, timing and type of vaccination in an emergency section setting [25]. Furthermore, a confounding factor on immunity against SARS-CoV-2 infection is the number of previous infections, which was not assessed. In addition, assembling broad and complete pieces of information in the emergency section of the medical records was difficult and complex because, due to COVID-19, the hospital facilities were under pressure. For this reason, many biomedical findings are missing.

## 5. Conclusions

The effects of Omicron and other COVID-19 variants on routine blood analyses are still under investigation, and ongoing research is essential to comprehensively explain the full spectrum of hematological and biochemical changes associated with SARS-CoV-2 variants. Healthcare professionals must remain vigilant in monitoring these parameters to tailor appropriate interventions and provide optimal care for individuals affected by Omicron COVID-19. Furthermore, the effects of COVID-19 on routine blood analyses are multifaceted, encompassing direct impacts on hematological and coagulation parameters, changes in patient behavior and alterations in healthcare delivery. As the situation is still evolving, the adaptation of diagnostic practices is essential to ensure the continued effectiveness of routine blood analyses in providing valuable insights into patient health. 

In conclusion, this research is a step further in the challenge to extricate early biomolecular markers of COVID-19 development. Moreover, it could also be beneficial to produce reports dealing with human disorders provoked by viral or bacterial infections, including other coronaviruses.

## Figures and Tables

**Table 1:** The number of routine analyses available for each group and considered for the statistical analyses.

	*Emergency*	*Hospital Ward*	*ICU*	*Deceased*
***N. of patients***	***180***	***205***	***25***	***35***
Creatinine	171	179	23	31
Azotemia	160	179	23	31
Red Blood Cells (RBCs)	178	205	25	35
Hemoglobin (Hb)	178	205	25	35
Hematocrit (Hct)	178	205	25	35
Mean Corpuscular Volume (MCV)	178	205	25	35
Mean Corpuscular Hemoglobin (MCH)	178	205	25	35
Mean Corpuscular Hemoglobin Concentration (MCHC)	178	205	25	35
Red Blood Cell Distribution Width (RDW)	178	205	25	35
Monocytes	178	205	25	35
Eosinophils	178	205	25	35
Basophils	178	205	25	35
White Blood Cells (WBCs)	178	205	25	35
Neutrophils	178	205	25	35
Lymphocytes	178	205	25	35
Platelets (PLTs)	178	205	25	35
Platelecrit (PCT)	178	205	25	35

**Table 2:** Recorded symptoms and comorbidities characterizing the recruited individuals for each group.

	*Emergency*		*Hospital Ward*		*ICU*		*Deceased*	
	*M (76)*	*F (104)*	*M (105)*	*F (100)*	*M (14)*	*F (11)*	*M (23)*	*F (12)*
***COVID-19 symptoms***								
***Fever***	30 (39.47%)	58 (55.77%)	55 (52.38%)	43 (43.00%)	5 (35.71%)	5 (45.45%)	11 (47.83%)	7 (58.33%)
***Cough***	26 (34.21%)	48 (46.15%)	36 (34.29%)	28 (28.00%)	1 (7.14%)	2 (18.18%)	8 (34.78%)	3 (25.00%)
***Dyspnea***	14 (18.42%)	30 (28.85%)	42 (40.00%)	31 (31.00%)	6 (42.86%)	6 (54.55%)	15 (65.22%)	9 (75.00%)
***Asthenia***	10 (13.16%)	23 (22.12%)	8 (7.62%)	12 (12.00%)	2 (14.29%)	3 (27.27%)	3 (13.04%)	2 (16.67%)
***Rhinitis***	6 (7.89%)	5 (4.81%)	6 (5.71%)	2 (2.00%)	0 (0.00%)	0 (0.00%)	0 (0.00%)	1 (8.33%)
***Memory deficits***	0 (0.00%)	0 (0.00%)	1 (0.95%)	0 (0.00%)	0 (0.00%)	0 (0.00%)	1 (4.35%)	1 (8.33%)
***Vertigo***	2 (2.63%)	3 (2.88%)	0 (0.00%)	4 (4.00%)	0 (0.00%)	0 (0.00%)	0 (0.00%)	0 (0.00%)
***Anosmia***	1 (1.32%)	2 (1.92%)	1 (0.95%)	4 (4.00%)	0 (0.00%)	0 (0.00%)	0 (0.00%)	1 (8.33%)
***Ageusia***	1 (1.32%)	2 (1.92%)	1 (0.95%)	2 (2.00%)	0 (0.00%)	0 (0.00%)	0 (0.00%)	1 (8.33%)
***Depression or anxiety***	3 (3.95%)	2 (1.92%)	1 (0.95%)	4 (4.00%)	1 (7.14%)	0 (0.00%)	0 (0.00%)	1 (8.33%)
***Brain fog***	1 (1.32%)	0 (0.00%)	1 (0.95%)	0 (0.00%)	0 (0.00%)	0 (0.00%)	1 (4.35%)	0 (0.00%)
***Epistaxis***	0 (0.00%)	0 (0.00%)	1 (0.95%)	0 (0.00%)	0 (0.00%)	0 (0.00%)	0 (0.00%)	1 (8.33%)
***Arthralgia or myalgia***	12 (15.79%)	32 (30.77%)	7 (6.67%)	7 (7.00%)	2 (14.29%)	1 (9.09%)	0 (0.00%)	2 (16.67%)
***Headache***	8 (10.53%)	14 (13.46%)	6 (5.71%)	9 (9.00%)	0 (0.00%)	1 (9.09%)	0 (0.00%)	0 (0.00%)
***Paresthesia***	3 (3.95%)	0 (0.00%)	0 (0.00%)	2 (2.00%)	1 (7.14%)	0 (0.00%)	0 (0.00%)	0 (0.00%)
***Sore throat***	11(14.47%)	4 (3.85%)	6 (5.71%)	8 (8.00%)	0 (0.00%)	0 (0.00%)	0 (0.00%)	0 (0.00%)
***Comorbidities***								
***Lung diseases***	8 (10.53%)	11 (10.58%)	12 (11.43%)	21 (21.00%)	4 (28.57%)	3 (27.27%)	2 (8.70%)	2 (16.67%)
***Cardiac diseases***	15 (19.74%)	22 (21.15%)	54 (51.43%)	54 (54.00%)	9 (64.29%)	6 (54.55%)	16 (69.57%)	10 (83.33%)
***Dyslipidemia***	2 (2.63%)	2 (1.92%)	11 (10.48%)	9 (9.00%)	2 (14.29%	0 (0.00%)	1 (4.35%)	1 (8.33%)
***Chronic Renal Failure***	0 (0.00%)	2 (1.92%)	11 (10.48%)	11 (11.00%)	2 (14.29%	1 (9.09%)	6 (26.09%)	2 (16.67%)
***Oncological diseases***	3 (3.95%)	12 (11.54%)	13 (12.38%	15 (15.00%)	1 (7.14%)	2 (18.18%)	9 (39.13%)	3 (2500%)
***Diabetes***	2 (2.63%)	2 (1.92%)	19 (18.10%)	18 (18.00%)	3 (21.43%)	2 (18.18%)	3 (13.04%)	2 (16.67%)
***Gastrointestinal diseases***	9 (11.84%)	8 (7.69%)	11 (10.48%)	10 (10.00%)	4 (28.57%)	2 (18.18%)	4 (17.39%)	3 (25.00%)
***Neurological or psychiatric diseases***	5 (6.58%)	12 (11.54%)	15 (14.29%)	22 (22.00%)	3 (21.43%	6 (54.55%)	8 (34.78%)	4 (33.33%)
***Urologic diseases***	5 (6.58%)	5 (4.81%)	8 (7.62%)	5 (5.00%)	3 (21.43%	1 (9.09%)	6 (26.09%)	0 (0.00%)
***Ophthalmological diseases***	0 (0.00%)	1 (0.96%)	3 (2.86%)	3 (3.00%)	1 (7.14%)	0 (0.00%)	0 (0.00%)	0 (0.00%)
***Immunological, rheumatological or hematological diseases***	7 (9.21%)	19 (18.27%)	16 (15.24%)	14 (14.00%)	1 (7.14%)	0 (0.00%)	4 (17.39%)	2 (16.67%)

**Table 3:** ANOVA data of the studied blood parameters for the four groups. *p*-values ≤ 0.05 are shown in bold.

Omicron COVID-19 Effect							
	dF	F-Value	*p*-Value		dF	F-Value	*p*-Value
**Creatinine**				**Monocytes**			
Outcome	3	5.500	**0.0010**	Outcome	3	2.626	**0.0500**
Sex	1	0.011	0.9178	Sex	1	0.060	0.8063
Outcome × Sex	3	0.265	0.8510	Outcome × Sex	3	0.486	0.6921
**Azotemia**				**Eosinophils**			
Outcome	3	26.175	**<0.0001**	Outcome	3	0.212	0.8881
Sex	1	0.756	0.3852	Sex	1	**0.002**	0.9690
Outcome × Sex	3	2.278	0.0792	Outcome × Sex	3	1.550	0.2008
**Red Blood Cells**				**Basophils**			
Outcome	3	11.878	**<0.0001**	Outcome	3	3.883	**0.0093**
Sex	1	7.523	**0.0063**	Sex	1	3.125	0.0778
Outcome × Sex	3	0.886	0.4483	Outcome × Sex	3	0.1936	0.1936
**Hemoglobin**				**White Blood Cells**			
Outcome	3	15.505	**<0.0001**	Outcome	3	1.323	0.2662
Sex	1	8.502	**0.0037**	Sex	1	0.521	0.4708
Outcome × Sex	3	0.1759	0.1759	Outcome × Sex	3	0.400	0.7534
**Hematocrit**				**Neutrophils**			
Outcome	3	7.957	**<0.0001**	Outcome	3	1.587	0.1918
Sex	1	11.825	**0.0006**	Sex	1	1.013	0.3147
Outcome × Sex	3	0.908	0.4372	Outcome × Sex	3	0.198	0.8975
**MCV**				**Lymphocytes**			
Outcome	3	0.434	0.7291	Outcome	3	0.721	0.5400
Sex	1	3.698	0.0551	Sex	1	**0.003**	0.9581
Outcome × Sex	3	4.356	**0.0049**	Outcome × Sex	3	0.105	0.9572
**MCH**				**Platelets**			
Outcome	3	0.734	0.5320	Outcome	3	2.041	0.1075
Sex	1	0.027	0.8688	Sex	1	5.742	**0.0170**
Outcome × Sex	3	2.057	0.1053	Outcome × Sex	3	1.994	0.1142
**MCHC**				**Platelecrit**			
Outcome	3	11.367	**<0.0001**	Outcome	3	0.593	0.6201
Sex	1	0.046	0.8299	Sex	1	3.057	0.811
Outcome × Sex	3	4.426	**0.0044**	Outcome × Sex	3	1.560	4.681
**RDW**							
Outcome	3	16.817	**<0.0001**				
Sex	1	4.079 × 10^−4^	0.9839				
Outcome × Sex	3	0.508	0.6772				

**Table 4:** AUC scores for the creatinine, azotemia, RBC, Hb, Hct, MCV, MCH, MCHC, RDW, monocytes, eosinophils, basophils, WBC, neutrophils, lymphocytes, PLT and PCT. The highest scores were found for creatinine, azotemia and RDW in the *deceased* group when compared with the patients in the *emergency section*. Significant scores are shown in bold.

	*Deceased* vs. *Emergency*		*ICU* vs. *Emergency*	
	AUC (Area under the Curve)	95% Confidence Interval	AUC (Area under the Curve)	95% Confidence Interval
Creatinine	**0.814**	0.719–0.909	0.699	0.573–0.824
Azotemia	**0.837**	0.728–0.945	0.757	0.622–0.893
RBC	0.748	0.639–0.857	0.761	0.668–0.854
Hb	0.738	0.631–0.844	0.764	0.656–0.873
Hct	0.712	0.596–0.828	0.752	0.631–0.872
MCV	0.479	0.356–0.603	0.53	0.393–0.667
MCH	0.467	0.338–0.595	0.616	0.494–0.737
MCHC	0.683	0.579–0.788	0.701	0.587–0.815
RDW	**0.872**	0.798–0.946	0.745	0.614–0.876
Monocytes	0.671	0.548–0.793	0.55	0.405–0.695
Eosinophils	0.707	0.594–0.821	0.548	0.42–0.676
Basophils	0.509	0.381–0.637	0.485	0.344–0.627
WBC	0.608	0.49–0.726	0.482	0.331–0.633
Neutrophils	0.704	0.6–0.809	0.518	0.362–0.674
Lymphocytes	0.666	0.56–0.772	0.625	0.503–0.748
PLT	0.685	0.58–0.79	0.531	0.396–0.665
PCT	0.624	0.517–0.732	0.53	0.395–0.664

**Table 5:** Positive predictive values (PPVs—probability that the patient has the condition when restricted to those patients who tested positive) in the *deceased*, *ICU* and *hospital ward* groups and negative predictive values (NPVs—probability that a patient who has a negative test result indeed does not have the condition) in the *emergency* group are based on the reference range values (out of range for PPV; in range for NPV) creatinine, azotemia, RBC (men and women), Hb (men and women), Hct, MCV, MCH, MCHC, RDW, monocytes, eosinophils, basophils, WBC, neutrophils, lymphocytes, PLT and PCT. Significant scores are shown in bold.

	PPV *Deceased*	PPV *ICU*	PPV *Ward*	NPV *Emergency*
Creatinine (0.8–1.2 mg/dL)	0.581	0.565	0.503	0.538
Azotemia (7–22 mg/dL)	0.742	0.478	0.246	**0.919**
RBC				
(Men 4.7–6.1 × 10^6^/µL)	0.783	0.714	0.495	0.760
(Women 4.2–5.4 × 10^6^/µL)	0.583	0.545	0.300	0.765
Hb				
(Men 14–18 g/dL)	0.739	0.643	0.495	**0.893**
(Women 12–16 g/dL)	0.333	0.455	0.270	**0.893**
Hct (38–52%)	0.629	0.560	0.332	**0.820**
MCV (80–100 fl/cell)	0.257	0.080	0.078	**0.904**
MCH (27–33 pg/cell)	0.400	0.080	0.161	**0.856**
MCHC (32–36 g/dL)	0.371	0.160	0.176	**0.906**
RDW (11.6–14.6%)	0.531	0.400	0.200	**0.928**
Monocytes (0.2–0.6 × 10^3^/µL)	0.486	0.440	0.332	**0.811**
Eosinophils (0.1–0.5 × 10^3^/µL)	**0.914**	0.577	0.737	0.383
Basophils (0–0.3 × 10^3^/µL)	0.086	0.000	0.044	**1.000**
WBC (4.4–11.3 × 10^3^/µL)	0.343	0.320	0.293	**0.839**
Neutrophils (1.8–7.7 × 10^3^/µL)	0.429	0.440	0.229	**0.883**
Lymphocytes (1.0–4.8 × 10^3^/µL)	0.743	0.440	0.498	0.711
PLT (1.5–4.0 × 10^5^/L)	0.286	0.160	0.244	**0.861**
PCT (0.12–0.36%)	0.743	0.480	0.593	0.539

**Table 6:** Spearman correlation values for the blood biomarkers and the patients’ outcome. Significant values are shown in bold.

Spearman’s Correlation		
	Spearman’s Rho	*p*-Value
Creatinine	0.295	**<0.001**
Azotemia	0.364	**<0.001**
RBC	−0.253	**<0.001**
Hb	−0.237	**<0.001**
Hct	−0.256	**<0.001**
MCV	0.023	0.632
MCH	−0.011	0.823
MCHC	−0.175	**<0.001**
RDW	0.335	**<0.001**
Monocytes	−0.035	0.458
Eosinophils	−0.177	**<0.001**
Basophils	−0.071	0.134
WBC	0.051	0.287
Neutrophils	0.102	0.032
Lymphocytes	−0.225	**<0.001**
PLT	−0.132	**<0.005**
PCT	−0.086	0.069

**Table 7:** Spearman correlations for the age parameter in the *deceased* group only. Significant values are shown in bold.

Spearman’s Correlation		
	Spearman’s Rho	*p*-Value
Creatinine	−0.095	0.602
Azotemia	0.268	0.141
RBC	men −0.154 women −0.098	men 0.469 women 0.735
Hb	men 0.021 women −0.444	men 0.923 women 0.124
Hct	men −0.056 women −0.254	men 0.791 women 0.378
MCV	0.091	0.595
MCH	0.062	0.716
MCHC	−0.126	0.464
RDW	0.244	0.174
Monocytes	0.140	0.414
Eosinophils	0.220	0.200
Basophils	0.109	0.536
WBC	0.526	**0.002**
Neutrophils	0.421	**0.014**
Lymphocytes	0.163	0.343
PLT	men 0.043 women −0.319	men 0.841 women 0.269
PCT	0.036	0.835

**Table 8:** The effects of vaccination on the analyzed biomarkers in a two-way ANOVA. The sex effect was not considered because it was not significant. Significant scores are shown in bold.

Omicron COVID-19 and Vaccination Effects									
	Vaccination (Yes/No)			Outcome			Vaccination × Outcome		
	dF	F-Value	*p*-Value	dF	F-Value	*p*-Value	dF	F-Value	*p*-Value
Creatinine	1	2.235	0.1358	3	3.675	0.0123	3	1.175	0.3189
Azotemia	1	1.775	0.1836	3	24.347	<0.0001	3	4.521	0.0039
Red Blood Cells	1	3.199	0.0744	3	7.573	<0.0001	3	0.449	0.7180
Hemoglobin	1	2.847	0.0922	3	10.767	<0.0001	3	3.900	0.0091
Hematocrit	1	0.005	0.9427	3	3.963	0.0083	3	0.994	0.3955
Mean Corpuscular Volume	1	0.049	0.8243	3	1.502	0.2133	3	4.559	0.0037
Mean Corpuscular Hemoglobin	1	0.659	0.4172	3	1.698	0.1666	3	2.937	0.331
Mean Corpuscular Hemoglobin Concentration	1	5.478	**0.0197**	3	13.598	<0.0001	3	7.725	<0.0001
Red Distribution Width	1	0.652	0.4199	3	15.820	<0.0001	3	0.380	0.7675
Monocytes	1	2.987	0.0846	3	1.553	0.2001	3	0.556	0.6446
Eosinophils	1	1.141	0.2861	3	0.480	0.6962	3	0.775	0.5086
Basophils	1	0.77	0.7809	3	5.646	0.0008	3	0.155	0.9263
White Blood Cells	1	2.893	0.0897	3	1.058	0.3668	3	0.737	0.5303
Neutrophils	1	1.686	0.1949	3	1.351	0.2574	3	0.173	0.9149
Lymphocytes	1	1.134	0.2876	3	0.415	0.7423	3	0.299	0.8258
Platelets	1	0.362	0.5476	3	1.168	0.3214	3	0.841	0.4718
Plateletcrit	1	0.135	0.7134	3	0.165	0.9197	3	0.835	0.4754

## Data Availability

Data are available upon request.
